# What’s your poison? Cyanide production regulated by a bHLH transcription factor in *Lotus japonicus*

**DOI:** 10.1093/jxb/erac031

**Published:** 2022-04-18

**Authors:** Viviana C Rosati

**Affiliations:** Department of Biology, Centre for Novel Agricultural Products (CNAP), University of York, Wentworth Way, York YO10 5DD, UK

**Keywords:** bHLH, cyanogenic glucosides, *Lotus japonicus*, methyl jasmonate, transcription factor

## Abstract

Humans hold a love–hate relationship with cyanogenic plants. Preferentially selected for domestication due to their heightened pest resistance, they can nevertheless threaten both human and livestock health if not consumed in moderation or adequately processed. Over 20% of crops are cyanogenic due to the stable accumulation of cyanogenic glucosides (CNglcs) and, while the biochemical pathways of these specialized metabolites are known, the same cannot be said regarding their underlying molecular pathways. In a comprehensive study, Chen *et al.* (2022) characterized a basic helix–loop–helix (bHLH) transcription factor that is responsive to methyl jasmonate and directly induces expression of the key CNglc biosynthetic gene in the model species *Lotus japonicus*.

## Teaser

This article comments on:

## Primary? Secondary? Both?

Plant metabolites are often placed into the discrete categories of primary or secondary. However, for CNglcs, that line is blurred as not only do they release hydrogen cyanide gas as a defence against herbivory, they are also involved in nitrogen transport and turnover, and physiological processes such as germination and bud burst (Gleadow and Møller, 2014; Pičmanová *et al.*, 2015; Del Cueto *et al.*, 2017; Bjarnholt *et al.*, 2018) (Box 1). The CNglc biosynthetic pathways are known in many cyanogenic species across different families and are both developmentally regulated (generally decreasing as tissues mature) and environmentally regulated (induced by factors such as drought or high nitrogen availability) (Gleadow and Møller, 2014). Whether herbivory can induce production of CNglcs is debatable as they are ultimately classed as phytoanticipins—already synthesized prior to attack by herbivores or pathogens. However, insect-feeding studies such as those undertaken in wild lima beans (*Phaseolus lunatus*) have seen marked increases in CNglc concentration in response to herbivore pressure (Ballhorn *et al.*, 2016).

Box 1. **Metabolism of cyanogenic glucosides**

Simplified representation of the cyanogenic glucoside biosynthesis, bioactivation, detoxification, and alternative turnover pathways found across cyanogenic plant species. The alternative turnover pathway occurs without the release of hydrogen cyanide gas (HCN), though both endogenous turnover pathways result in nitrogen being recaptured as ammonia which may then be used in primary metabolism. The endogenous turnover pathways clearly illustrate why cyanogenic glucosides are not merely defence compounds but play dual roles in both primary and secondary metabolism, and why acyanogenic or low-cyanogenic mutants often show growth deficits in comparison with wild-type plants.

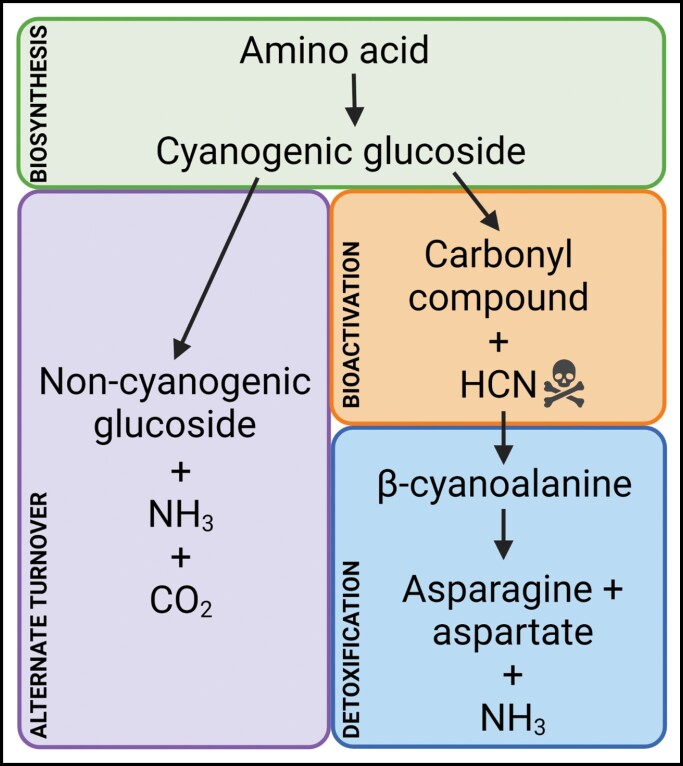

Previous studies have attempted to discern the hormonal pathways controlling fluctuations in cyanide potential in response to environmental factors. Salicylic acid and methyl jasmonate have been implicated in the increase of CNglc concentrations in species such as sorghum (*Sorghum bicolor*) (Zhu-Salzman *et al.*, 2004) although other studies observed no such change in response to the exogenous application of these hormones (Sohail *et al.*, 2020). The age of plants tested may change the results linking hormone application to cyanide potential. For example, as young sorghum seedlings produce maximum amounts of the CNglc dhurrin, it is possible that concentrations are less likely to be induced further by herbivory, while in older plants this would not hold true. Fluctuations in CNglc concentrations are often found to be regulated at the transcriptional level (Busk and Møller, 2002; Nielsen *et al.*, 2016), though the molecular pathways linking hormone signalling to increases in expression of CNglc biosynthetic genes are not known. The study by Chen *et al.* (2022) is one of the first to bridge these gaps.

Chen *et al.* (2022) initially showed that herbivore pressure by *Plutella xylostella* larvae increased the concentration of the CNglc lotaustralin due to increased expression of the three CNglc biosynthetic genes in *L. japonicus*. With the hypothesis that methyl jasmonate was responsible for the observed increase, exogenous application of the hormone was then tested. It was found that this also produced an increase in lotaustralin concentration and in the expression of genes involved in jasmonate synthesis. At this point, the study may have concluded; however, Chen *et al.* (2022) went further, identifying the bHLH transcription factor LjbHLH7 [a homologue of *Arabidopsis thaliana* AtMYC2, which plays a pivotal role in jasmonate signalling (Kazan and Manners, 2013)] not only as responsive to methyl jasmonate, but also as being able to bind directly to two G-Box promoter motifs in the *CYP79D3* gene of *L. japonicus*. Given that CYP79D3 is the first and rate-limiting enzyme of lotaustralin biosynthesis in the plant, any increase or decrease in expression is likely to correspond to a relative change in CNglc concentration. Chen *et al.* (2022) continue with this line of evidence, finding that transgenic *L. japonicus* plants overexpressing *LjbHLH7* have both higher CNglc content and enhanced insect resistance. The untangling of the regulatory network is also underway, with the JASMONATE-ZIM DOMAIN protein LjJAZ4 identified as a repressor of LjbHLH7 transcriptional activity via direct binding (Box 2).

Box 2. **Cyanogenic glucoside regulation by bHLH transcription factors**

In the new model proposed by Chen *et al.* (2022), insect feeding increases jasmonate synthesis in *Lotus japonicus*, with high jasmonate concentrations inducing expression of basic helix–loop–helix (bHLH) transcription factors and repressing JASMONATE-ZIM DOMAIN (JAZ) proteins. JAZ proteins bind directly to bHLH transcription factors and repress their function, but in the presence of jasmonates they can be targeted for degradation by the SCF^COI1^ ubiquitin ligase complex (Pauwels and Goossens, 2011). bHLH transcription factors bind to G-Box motifs in the promoter of the key cyanogenic glucoside (CNglc) biosynthetic gene that codes for a cytochrome P450 enzyme (CYP79). This induces CNglc production and, upon tissue disruption, such as feeding by insect larvae, the CNglcs are broken down to release hydrogen cyanide gas. In turn, the hydrogen cyanide gas is toxic to pests and inhibits their feeding.

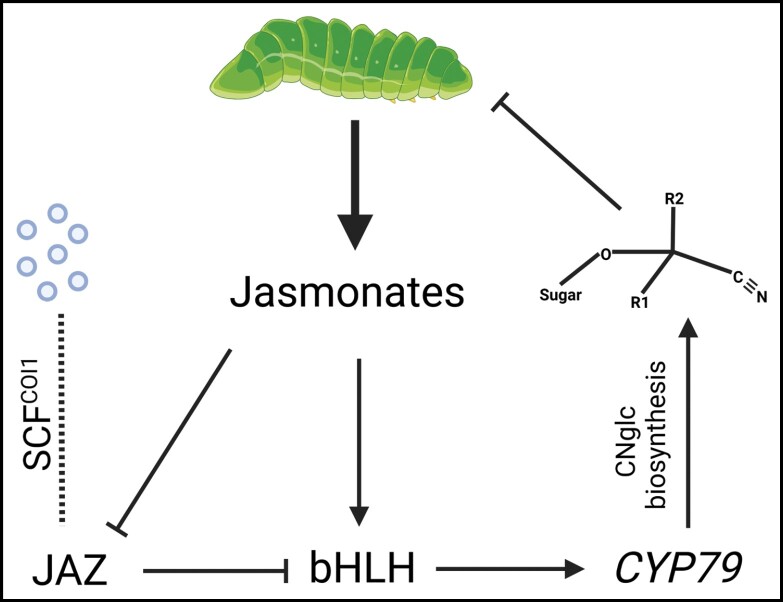

Although it cannot be assumed that regulatory factors will be similar across species given that the cyanogenesis genes between species are non-homologous (arising in phylogenetically unrelated plants via convergent evolution), the identification of these regulatory factors by Chen *et al.* (2022) is a promising start in what is a model species for cyanogenic plants. Jasmonates are widely seen to play essential roles in secondary metabolite regulation in response to mechanical damage, herbivory, and pathogens, and have specifically been found to increase CNglc concentration in other cyanogenic species such as wild lima beans (Kautz *et al.*, 2014). Furthermore, bHLH transcription factors have also been implicated in CNglc production in almonds (*Prunus dulcis*) (Sánchez-Pérez *et al.*, 2019).

## New possibilities for the regulation of cyanogenic glucosides

The challenge facing the use of many cyanogenic species in modern agriculture is this: while humans and livestock are susceptible to the effects of cyanide, reduction or removal of CNglcs may increase susceptibility to pests and also negatively affect plant growth. This has been observed in cyanogenic species such as cassava (*Manihot esculenta*), sorghum, and rubber trees (*Hevea brasiliensis*), where the production of low-cyanogenic or acyanogenic lines resulted in reduced growth rates and yields (Jørgensen *et al.*, 2005; Kongsawadworakul *et al.*, 2009; Blomstedt *et al.*, 2018). Another factor for consideration is that affecting developmental regulation of CNglcs to produce low-cyanogenic lines may see no effect on environmental regulation, with mutant plants reaching the same cyanide potential as that of the wild type when stressed (Rosati *et al.*, 2019).

To date, much of the focus has been on knocking out CNglcs in their entirety via targeting of the first enzyme in the CNglc biosynthetic pathway (Jørgensen *et al.*, 2005; Blomstedt *et al.*, 2012). Elucidating regulatory molecules is pivotal to provide new targets that do not involve the complete elimination of CNglcs, or that target environmental but not developmental regulation, or vice versa. Targeting environmental but not developmental CNglc regulation would enable cyanogenic species to maintain protection against pests during early development but would avoid environmental increases that prevent farmers from being able to safely use their forage crops, or that increase poisoning risks for people with diets high in cyanogenic species such as cassava. Alternatively, targets that enable higher concentrations of CNglcs to accumulate in plant tissues could be beneficial in biofuel crops, where high levels of sugars are desirable, or in crops exposed to elevated levels of pest pressure.

The benefits of novel targets for CNglc regulation are exemplified in almonds, where wild, bitter lines accumulate high concentrations of CNglcs, whereas domestic lines do not. This has eventuated from the selection of plants carrying a single base pair mutation in the bHLH transcription factor gene, *bHLH2*, which prevents the protein from forming a functional dimer and activating transcription of the two prunasin biosynthetic genes *PdCYP79D16* and *PdCYP71AN24* (Sánchez-Pérez *et al.*, 2019). With the advent of gene editing, and the relative ease of creating single base pair mutations in plant genomes, it is possible to engineer small genomic changes that produce drastic phenotypic effects, such as changing a toxic, inedible species to one that is a safe and nutritious part of the diet of millions of people across the globe—we only need to know which genes to target.
